# Adolescent health and adaptation in Canada: examination of gender and age aspects of the healthy immigrant effect

**DOI:** 10.1186/s12939-014-0103-5

**Published:** 2014-11-14

**Authors:** Kyunghwa Kwak, Floyd Rudmin

**Affiliations:** Division of Mental Health, Norwegian Institute of Public Health, Oslo, Norway; Centre for Research on Migration, Refugees, and Belonging, School of Law and Social Sciences, University of East London, London, UK; Department of Psychology, UIT Norway’s Arctic University, Tromsø, N-9037 Norway

**Keywords:** Adolescents, Health and adaptation, Healthy immigrant effect, Acculturation

## Abstract

**Introduction:**

A longstanding and widely held assumption is that immigrants suffer from ill health and adaptation problems. Yet recent studies show that immigrants report the same or better state of health compared to their native-born counterparts. This phenomenon, known as the *healthy immigrant effect*, has been found in studies of specific health conditions of adults. The present study focuses instead on adolescents and extends its examination of the healthy immigrant effect, measuring both health and adaptation.

**Methods:**

Using data from population samples in the Canadian Community Health Survey (2007), foreign-born immigrant adolescents (n = 920) were compared to non-immigrant adolescents (n = 13,572) for their self-report to questionnaire items for health (general health, mental health, chronic illnesses with psychosomatic symptoms, and psychological illnesses) and adaptation (daily life stress, life satisfaction, and sense of belonging). Adolescents’ gender, age, and length of residence were analyzed for the effects.

**Results:**

Immigrant adolescents were better than non-immigrant peers on the four health measures, and did not differ from non-immigrants on the three adaptation measures despite having less household income and more family members in the household. Immigrant girls exhibited more resilient adaptability, while young immigrant boys and older non-immigrant girls displayed some potential vulnerability. Length of residence, on the other hand, did not contribute to differences for the health and adaptation of immigrant adolescents.

**Conclusions:**

The healthy immigrant effect was confirmed in a community population sample of adolescents in Canada. Foreign-born immigrant adolescents experience better health, as well as good adaptation equal to their native-born peers. These outcomes call for further research on sustaining good health and adaptation of the immigrant population, in particular by providing age-related effective services and prevention strategies.

## Introduction

The *healthy immigrant effect* describes an initial advantage of immigrant health status which is lost over time as the length of residence increases in the settlement society [1]. A growing body of medical and social science evidence has shown that immigrants are indistinguishable from, or superior to, non-immigrants on measures of health and well-being [1–6]. Although more attention has been paid to examine this phenomenon with adult immigrant populations, studies investigating the healthy immigrant effect with children and adolescents of immigrant families still remain scarce [1,7].

By comparing foreign-born immigrant adolescents and their native-born peers, the present study elucidates two research questions concerning the well-being of adolescents. The first is whether adolescents’ self-reports of health and adaptation will yield comparable outcomes to adult populations in demonstrating immigrants’ health being better than the health of their native-born counterparts [1,5]. The second is to what extent the healthy immigrant effect can be differentiated in terms of gender and age for adolescents [8–11].

The present study, acknowledging the interlink of mental health and physical symptoms [12], focuses on adolescents’ well-being to ascertain the healthy immigrant effect by examining physical, mental and psychological domains of health and daily stress, life satisfaction, and sense of belonging for adaptation domains. It is possible that immigrant experience may not be adverse to their health and adaptation; and in turn, to challenge the tradition of conceiving immigrants as suffering from ill health and maladaptation which is reported in the literature as the “*immigrant paradox*” [13–16] or “*epidemiological paradox*” [17,18].

The health of immigrant adolescents needs to be understood with the influence of social determinants such as their socioeconomic status [19] and resilience [20–22]. Even though immigrant adolescents have disadvantaged and less affluent surroundings in general, [13] in Canada their mental health advantages over their Canadian native-born counterparts were affected less by family dysfunction or negative family factors [23]. So, while family affluence has shown positive influences as a material condition affecting individuals’ health behaviors and parental support, as opposed to poverty linking to negative consequences, [13,24] family affluence itself may not affect and explain the health conditions of immigrant children and adolescents. Instead, declines in immigrants’ initial advantages in health have been explained by changes in the immigrant family lifestyle. These changes increase with length of residence in the settlement society as immigrants are exposed to and engage with the new cultural and socialization standards (e.g., in the US, the Hispanic immigrant groups [3]; Asian and Pacific Islander groups [4]).

### Adolescent health and adaptation

People in good health display more than an absence of negative physical symptoms; they also display positive adaptation, a sense of control of their social surroundings, life satisfaction, and engagement with others [25]. One of the most robust predictors of adolescents’ health is their perception of the level of stress they experience in terms of chronic strain and daily hassles [26]. Accordingly, to be healthy, adolescents need to improve the conditions of their daily life and to build a sense of cohesion in the community [7]. Therefore, it is essential and appropriate to examine the health and adaptation of adolescents together when considering the issues of their overall well-being.

In an evaluation of immigrant adolescents’ overall well-being, it is important to trace their developmental, social, and familial aspects of adaptation using a framework that compares them with their national-group peers in the settlement society [27]. Compared to their native counterparts, immigrant adolescents generally experience more daily hassles and stress related to parents and school [28]. Although their familial surroundings can be a source of stress, they can also be a protective beneficial ground for good adaptation because adolescents’ coping and adaptation are supported by family relations, and family relations in turn serve as a stress-buffer mechanism, easing alienation and ensuring a sense of security [10,29,30]. So, it is possible to find that the lives of immigrant youths with their experience as immigrants per se do not necessarily lead to adaptation problems [16].

Immigrant adaptation is the process through which individuals seek to satisfy their needs, pursue their goals and manage demands encountered after relocating to a new society [31]. Consequently, immigrants need to possess functional abilities to perform new and redefined roles in the settlement society at productive and satisfactory levels [32]. Based on such two aspects in their experiences, managing their present conditions and preparing for their future, adolescent immigrants have more diverse contexts of support than adult immigrants as they seek to build social capital. Since immigrant adolescents, as compared to adults, are in a better position to access a range of resources through social relationships and education, they may be also better able to develop skills for participation and to enhance their means of access into the new society. With both institutional support and personal social networks, immigrant adolescents have opportunities to develop their psychological stability and confidence, the results of which are cumulative at multiple levels [31,33].

A research question is, then, whether the outcomes of immigrant adolescent health and adaptation would be comparable to those of immigrant adults. For example, immigrants’ somatization, a mechanism by which an individual expresses psychological distress with physical symptoms, has been explained as related to their social isolation in times of stress [34]. However, the prevalence of psychosomatic symptoms between immigrant and non-immigrant adult populations has been reported as very similar in clinical samples, [35] with no differences found by gender and age, [36] or by ethnocultural background [37]. Overall, for adult immigrants, level of stress, sense of belonging, and degree of life satisfaction are important measures of adaptation, all of which have been associated with their somatization [38].

In the present study, three specific points are considered with regard to how adolescents’ self-perception of health and adaptation needs to be studied for the healthy immigrant effect. Firstly, a large nationally representative data set are needed across all regions of a nation to avoid the possibility of yielding outcomes affected by the small sample size or by the data collection from the areas where immigrant populations are concentrated [10,12]. Secondly, research yields that adolescent self-reporting is considered to be accurate and reliable (e.g., greater associations between stressors and symptoms in the adolescents’ own reports than by others [26]; large discrepancies in reporting of the adolescent symptoms and behavioral problems by parents and by teachers [11,39]; high reliability and validity of self-assessed health by adolescents, [40,41]) while the validity and reliability of the instruments currently available to measure adolescents’ wellness are inadequate [42]. Thirdly, in general, research shows that adolescents’ self-reported health varies with social determinants such as socioeconomic status, material deprivation and parenting influenced by poverty, [8,10,43–45]; therefore, a comparative study of health with immigrant and non-immigrant adolescents should control for such social determinants.

### Effects of gender and age during adolescence

The differences exhibited by adolescent girls and boys in health and adaptation have been reported as salient; girls are more susceptible and vulnerable, [8,46] similar to the adult populations [47]. Such generalisability is supported by the ethnic equivalence theory which posits certain degrees of similarities across ethnic groups [48,49]. On the other hand, the common vulnerability theory and the coping deficit theory have been questioned for explaining gender differences by suggesting girls’ higher vulnerability and susceptibility to their social context and their lack of coping skills, which in turn leads to experiencing more symptoms [50].

The primary source of the social context affecting adolescent mental health is the home environment [12,51]. At home, immigrant girls may live in less favorable familial contexts, such as being involved in stress-provoking situations arising from their parents’ gender differential parenting practices [19]. As such, they may exhibit more psychosomatic symptoms than boys [14]. Immigrant boys, on the other hand, are more vulnerable in the restricted urban socialization context, [52] and tend to externalize their problems in engaging in risky behaviors and delinquency [19].

Although adolescents’ self-perception of health and adaptation vary with their age as well as gender, effects of age have been studied much less than those of gender [11]. Since the two most frequently experienced symptoms of psychological illness by adolescents are of anxiety and mood disorders, with considerable gender and age differences, [12,51,53–55] this study also investigates to what extent immigrant adolescents are thought to be different in their self-reporting of these symptoms compared to their non-immigrant peers. So far, studies have shown that significantly more girls experience the symptoms of the anxiety and mood disorders although no difference has been found in the average age of the onset between gender, [12] or by SES, [55] or by ethnicity [56]; but it is an early stage of research on psychological illnesses in terms of immigrant status [57].

Studies with large sample sizes suggest that there is still a need to clarify how the interplay of gender, age, and immigrant status is to be found with adolescents for their perception of well-being. During early adolescence, boys (ages 11 to 13 years) have shown better overall mental health, [9] while reports of subjective health complaints among young adolescents (11, 13 and 15-year-old) increased with age more so for girls than boys [46]. When compared by immigrant status, young immigrant adolescents (11–15 years old) in Italy reported more psychosomatic symptoms and lower life satisfaction than their native classmates, but the differences between immigrant and non-immigrant groups were not explained by SES, or lack of social integration [58]. However, with a large data set of grades 7 to 12 students in the US, Harker [10] revealed that the first-generation immigrant adolescents experienced more positive well-being than their native-born counterparts of similar demographic and family backgrounds, which was sustained by protective factors from family and community, such as parental supervision and lack of parent–child conflict. Therefore, it is critical to investigate how particular age groups of adolescents in a particular society would show different degrees influenced by various factors contributing to their well-being [59].

In sum, the present study seeks to examine the healthy immigrant effect in the Canadian adolescent population. The first objective is to investigate whether immigrant adolescents’ reports are better than their non-immigrant peers; if so, to what extent the healthy immigrant effect is evident in the domains of health (measured as general health, mental health, chronic illnesses, and psychological illness) and in the domain of adaptation (measured as daily stress, life-satisfaction, and sense of belonging). The second objective is to clarify the influences of gender and age in conjunction with the healthy immigrant effect. Immigrant adolescents are predicted to show resilience by exhibiting better health and robust adaptation. Girls may show vulnerability in their experiences; however, it is still possible to find no gender difference, if they show good social adaptability regardless of their less favorable self-reporting of health or of immigrant status. In terms of age, this study hypothesizes that older adolescents will experience more health and adaptation problems, but it is not certain what changes or patterns would be shown in terms of the interactions with gender and immigrant status. The third objective is to detect differential influence by length of residence on immigrant adolescents’ perceptions of health and adaptation, in order to shed some light on the general trajectory of the healthy immigrant effect.

## Methods

### Data source

The data analysed for this study are from the Canadian Community Health Survey (CCHS Cycle 4.1) which was collected from January to December 2007 throughout all regions of Canada (Statistics Canada, 2012) [60]. The authors of the present study were not involved in questionnaire design or data collection; consequently, obtaining consent from the participants and ethical approval were not the authors’ responsibilities.

For the purposes of the present study, participants aged 12–19 who were either foreign-born immigrant adolescents (N = 920) or Canadian native-born non-immigrant adolescents (N = 13,572) were selected. Among the immigrant adolescents, 625 had less than ten years of residence in Canada and 295 had ten years or more. CCHS sampling included randomly selected households from the lists of regional health units and from the lists of household telephone numbers with a target number proportional to the general population size of each region. Approximately half of the participants were interviewed in person; the other half were interviewed over the telephone. Participation in the survey was voluntary, and the overall national response rate was 78%, ranging from 75% to 86% by region. The respondents had the option of using English or French, or using one of 22 ethnic languages. The participants’ report on their “cultural and racial backgrounds” was used to derive their visible minority status in this study, and coded as 0 = White and 1 = non-White.

The demographic measures included for this study from the data source and their differences between the two immigrant-status groups are shown in Table 1. The mean age of the immigrant adolescents was slightly older. Slightly more boys (55% & 51% respectively) than girls (45% & 49%) participated in both immigrant and non-immigrant groups. Greater proportions of immigrant adolescents were visible minority members (71%) than non-immigrants (18%). Immigrant adolescents also reported higher numbers of family members living together in the household but lower levels of household income than did their non-immigrant peers. On the other hand, the participation ratios of adolescents with a parent were very similar, 76-78%, between immigrant and non-immigrant groups, as well as between boys and girls. The statistical analyses were carried out with Statistica 5.1.

**Table 1:** **Demographic measures for immigrant versus non-immigrant adolescents divided by gender**

**Measures (range)**	**Gender**	**Immigrants (N = 920)**		**Non-immigrants (N = 13572)**		**F-test (p < .05)**
		**M (SD)**	**N**	**M (SD)**	**N**	
Age (12–19)	female	15.52 (2.19)	412	15.46 (2.15)	6742	I > NI F ≈ M
	male	15.76 (2.16)	508	15.42 (2.14)	6830	no INT
Visible minority (0 or 1)	female	73 (%) (.44)	404	18 (%) (.39)	6473	I > NI F ≈ M
	male	69 (%) (.46)	500	18 (%) (.38)	6579	no INT
Household income (1–4)	female	3.03 (1.44)	316	3.69 (1.37)	5005	I < NI F < M
	male	3.28 (1.36)	362	3.78 (1.33)	5203	no INT
Household size (1–5)	female	3.84 (.96)	412	3.71 (.95)	6738	I > NI F ≈ M
	male	3.80 (.99)	508	3.74 (.95)	6826	no INT
Presence of a parent (0 or 1)	female	77 (%) (.42)	404	78 (%) (.41)	6607	I ≈ NI F ≈ M
	male	76 (%) (.43)	502	77 (%) (.42)	6692	no INT

(Source: Data from the Canadian Community Health Survey, 2007).

Notes:

(1) Visible Minority: 0 indicates white Caucasians; 1 indicates non-whites.

(2) Household Income: 1 = $0-$19,999, 2 = $20,000-$39,999, 3 = $40,000-$59,999, 4 = $60,000-$79,999, 5 = $80,000 or more.

(3) Household Size: 1 = 1 person, 2 = 2 persons, 3 = 3 persons, 4 = 4 persons, 5 = 5 or more persons.

(4) Presence of a Parent: 0 indicates without a parent; 1 indicates with a parent.

(5) I = immigrants, NI = non-immigrants; < or > means significant differences; ≈ means no differences.

### Measures

The four measures of health (general health, mental health, chronic illnesses with psychosomatic symptoms, and psychological illnesses) and three measures of adaptation (daily life stress, life satisfaction, and sense of belonging) were adopted from the CCHS (2007). The question formats and response scales were, for example, the single item for general health asked, “*In general, would you say your health is…..?*” with a response option ranging from 1 = *excellent* to 5 = *poor*, and the item for daily life stress asked, “*About the amount of stress in your life, would you say that most days are …..?*” with a response option ranging from 1 = *not at all stressful* to 5 = *extremely stressful*.

Participants were also asked whether or not they had had specific illnesses lasting six months in duration or longer: the two categories of illness were (1) chronic illnesses with psychosomatic symptoms (asthma, back problems, bowel disorders, migraine, and ulcers), and (2) psychological illnesses (mood disorders and anxiety disorders). With each condition coded as 0 = no and 1 = yes, the illness categories were computed by summing the binary coding. Consequently, chronic illnesses with psychosomatic symptoms ranged from 0 to 5, and psychological illnesses ranged from 0 to 2.

## Results

The first sets of analyses were carried out with the immigrant adolescents, comparing those with less than ten years of residence (N = 625) to those with ten years or more (N = 295). No significant differences were found for either health or adaptation measures, with or without the set of control variables as covariates (age, gender, visible minority status, household income, household size, and presence of a parent during the interview). Consequently, further analyses were done with all immigrant adolescents together as one group when they were compared to the non-immigrant adolescents.

The adolescents examined in this study were in good health and well-adapted according to their own self-reports. Table 2 shows means, standard deviations, and computational N’s for the four measures of health and three measures of adaptation with *lower* scores indicating healthier and better outcomes. The means for immigrant and non-immigrant adolescents were both below the scale midpoint for all four measures of health as well as two of the three adaptation measure showing self-perceptions of being healthy and of being well-adapted. For the one exception, sense of belonging, the mean scores were slightly above the scale midpoint, suggesting that both groups of adolescents had a mildly weak sense of belonging to their community.

**Table 2:** **Mean differences in health and adaptation measures between immigrant and non-immigrant adolescents by gender**

**Measures of health and adaptation**	**Gender**	**Immigrants**		**Non-immigrants**		**F-test (p < .05)**
		**M (SD)**	**N**	**M (SD)**	**N**	
**General health**						
*In general, would you say your health is.....?*	f	2.07 (.82)	306	2.18 (.83)	4844	I < NI F ≈ M
1 = *excellent* to 5 = *poor*	m	1.99 (.84)	355	2.11 (.84)	5035	no INT
**Mental health**						
*In general, would you say your mental health is.....?*	f	1.86 (.89)	295	1.87 (.87)	4653	I < NI F ≈ M
1 = *excellent* to 5 = *poor*	m	1.76 (.82)	344	1.85 (.85)	4779	no INT
**Chronic illnesses with psychosomatic symptoms**						
*Asthma, migraine, back problems, ulcer, & bowel disorders* (*Sum of 5 answers*)	f	0.24 (.51)	304	0.37 (.63)	4872	I < NI F > M
0 = *No,* 1 = *Yes*	m	0.14 (.36)	354	0.29 (.55)	4989	no INT
**Psychological illnesses**						
*Mood disorders & anxiety disorders* (*Sum of 2 answers*)	f	0.05 (.24)	305	0.10 (.36)	4827	I < NI F ≈ M
0 = *No,* 1 = *Yes*	m	0.03 (.20)	352	0.06 (.28)	5019	no INT
**Daily life stress**						
*About the amount of stress in your life, would you say that most days are....?*	f	2.74 (.99)	182	2.83 (.86)	2805	I ≈ NI F > M
1 = *not at all stressful to* 5 = *extremely stressful*	m	2.55 (1.00)	233	2.51 (.90)	2914	no INT
**Life satisfaction**						
*How satisfied are you with your life in general?*	f	1.68 (.70)	295	1.63 (.64)	4652	I ≈ NI F ≈ M
1 = *very satisfied to* 5 = *very dissatisfied*	m	1.68 (.58)	342	1.59 (.60)	4779	no INT
**Sense of belonging**						
*How would you describe your sense of belonging to your local community?*	f	2.03 (.74)	293	2.06 (.76)	4607	I ≈ NI F < M
1 = *very strong to* 4 = *very weak*	m	2.19 (.75)	341	2.08 (.76)	4734	sig INT

(Source: Data from the Canadian Community Health Survey 2007).

Notes: I = immigrants, NI = non-immigrants; < or > means significant differences; ≈ means no differences, controlling age, visible minority status, household income, household size, and presence of parent during the interview as covariates.

Nevertheless, of the positive outcomes of health and adaptation for both immigrant and non-immigrant adolescents, the F-tests using statistical significance levels of p < .05 shown in Table 2 revealed that the immigrant adolescents’ self-reports were still healthier than their non-immigrant peers on all four health measures -- general health, mental health, chronic illnesses with psychosomatic symptoms, and psychological illness, after controlling the covariate effects of age, visible minority status, household income, household size, and presence of a parent during the interview. Only chronic illness with psychosomatic symptoms showed a gender difference since girls reported experiencing more symptoms regardless of their immigrant status.

For adaptation, the adolescents were indistinguishable by immigrant status for all three measures of them - daily life stress, life satisfaction, as well as sense of belonging (see Table 2). Regardless of their immigrant status, all adolescents in this study perceived themselves as not being much stressed and fairly satisfied with their lives, although having a mildly weak sense of belonging to their community. This similar outcome between the two groups of adolescents suggests that the experience of the foreign-born immigrant adolescents had not adversely influenced them despite their less affluential family and household context (see Table 1). In terms of gender, girls, regardless of their immigrant status, reported more daily stress, whereas boys reported a weaker sense of belonging, but there was no gender difference in their life satisfaction. One significant interaction indicated immigrant boys, in particular, feeling a weaker sense of belonging to the community.

In order to consolidate the measures reported in Table 2, a Health Index and an Adaptation Index were computed. Table 3 presents the adolescents’ self-reports of their health and adaption by immigrant status, gender and age group. To compute the health and adaptation indices in Table 3, two steps were involved. First, the measures were transformed from their responses on the CCHS ranges to a 0.00 to 1.00 range. For example, a score of 2 on the General Health item became 0.25 representing a response of one-fourth of the 1–5 range. Second, the four transformed health measures were averaged to compute the Health Index, and the three adaptation measures were averaged to compute the Adaptation Index.

**Table 3:** **Differences in the indices of health and adaptation by immigrant status, gender and age**

**Measurement index**	**Age group**	**Immigrants**		**Non-immigrants**		**F-test p**
		**Female**	**Male**	**Female**	**Male**	
		**M (SD)**	**M (SD)**	**M (SD)**	**M (SD)**	
**Health index**	Young Teens	M = .12 (.08)	M = .12 (.08)	M = .14 (.10)	M = .14 (.09)	**Age** p < .05
		N = 116	N = 115	N = 1898	N = 1939	
	Mid Teens	M = .15 (.12)	M = .12 (.10)	M = .16 (.12)	M = .14 (.11)	**Gender** p < .001
		N = 107	N = 132	N = 1742	N = 1780	**Immigrant Status** p < .001
	Old Teens	M = .15 (.12)	M = .12 (.10)	M = .18 (.14)	M = .15 (.11)	**No INT**
		N = 71	N = 94	N = 965	N = 1009	
	Ns	Total N =635		N = 9333		
**Adaptation index**	Young Teens	M = .17 (.13)	M = .20 (.12)	M = .17 (.12)	M = .17 (.12)	**Age** p < .001
		N = 114	N = 115	N = 1895	N = 1944	
	Mid Teens	M = .30 (.14)	M = .28 (.13)	M = .30 (.12)	M = .27 (.13)	**Age x Gender** p < .01
		N = 107	N = 133	N = 1756	N = 1795	
	Old Teens	M = .31 (.16)	M = .31 (.12)	M = .33 (.13)	M = .30 (.13)	**Immigrant Status x Gender** p < .01
		N = 72	N = 94	N = 969	N = 1011	
	Ns	Total N = 635		Total N = 9370		

Notes:

(1) The Health index includes the measures of General Health, Mental Health, Chronic Illnesses with Psychosomatic Symptoms, Psychological Illnesses; The Adaptation Index includes those of Daily Life Stress, Life Satisfaction, and Sense of Belonging. Visible minority status, household income, household size, and presence of parent during the interview were controlled as covariates.

(2) Young Teens (12–14), Mid Teens (15–17), Old Teens (18–19 years old) inclusive.

ANCOVA analysis was carried out, controlling for visible minority status, household income, household size, and parent presence during the interview as covariates. For the Health Index, age, gender, and immigrant status all revealed significant effects without any interactions. For the Adaptation Index, two significant interactions were found: age by gender and gender by immigrant status, in addition to the age effect. The adolescents were significantly different across the three age groups -- young (aged 12–14), mid-aged (aged 15–17), and old adolescents (aged 18–19) -- the youngest group reporting the least problems of health or adaptation. The significant interactions of adaptation, age by gender and gender by immigrant status, suggest that although the older teens perceived themselves as less well-adapted, the magnitude of such increases with age was greater with girls, and this gender gap was greater in the non-immigrant groups.

Overall, findings can be summarized as: (1) immigrant adolescents were healthier without showing adaptation problems as compared to their native-born non-immigrant counterparts; (2) girls from both immigrant and non-immigrant groups reported experiencing more stress and chronic psychosomatic illnesses; however, their reports on psychological illness and life satisfaction were not different from those of boys; (3) adolescent boys regardless of their immigrant status felt a weaker sense of belonging to community, especially early teen immigrant boys; (4) in general, older adolescents reported more problems with health as well as with adaptation, but the two potentially vulnerable cohorts were older non-immigrant girls and younger immigrant boys, both of whom reported feeling less well adapted; and (5) immigrant adolescents did not report their well-being differently with their length of residence.

## Discussion

To understand the overall well-being of adolescents, the present study pursued two lines of inquiry. The first was to posit whether foreign-born immigrant adolescents’ perceptions of their health and adaptation were better than their non-immigrant peers, thus providing evidence to support the healthy immigrant effect in relation to length of residence. The second was to clarify the influences of gender and age in line with the healthy immigrant effect.

The findings of this study showed that foreign-born immigrant adolescents had not only better health conditions but also equally good adaptation when compared to their native-born non-immigrant counterparts in the Canadian general population. However, among the immigrant adolescents themselves, their self-reports of health and adaptation did not differ with length of residence. Thus, in this study the trajectory of immigrant adolescents’ well-being does not clearly accord with the fuller description of the healthy immigrant effect observed for adults – namely, that among immigrant adults, earlier advantages of health diminish as length of residence increases.

With regards to the influence of gender, the hypothesis predicting girls’ less effective adaptability was not supported in this study. Although the girls, regardless of immigrant status, reported experiencing more chronic psychosomatic symptoms and daily life stress, they also showed equally healthy self-reporting on the other measures of health and adaptation, as compared to boys. Hence, this set of findings provides some evidence not in accordance with the theoretical explanations that girls are more vulnerable and utilize less effective coping strategies to lessen or ease their stress and internalization of difficulties as suggested by the common vulnerability theory and coping deficit theory, for example [8,46,48].

Moreover, immigrant girls are to be credited for their healthier acculturation - a more robust social adaptability. Even though no measure was adopted to detect social adaptability in this study, it seems that immigrant girls are developing new supportive relationships more easily in their social surroundings and receive benefits from them for their adaptation [61] in spite of differential parenting practices by immigrant parents making them experience higher stress at home [19]. Their less affluent family environments also do not deter their adaptation; so, the social adaptability generated by the immigrant girls can be interpreted as their resilience entailing the use of internal protective factors (individual assets) in combination with external protective factors (resources from the immediate social surroundings) [21,22,52].

Considering age and gender together, older, girls, and non-immigrant adolescents perceived their health conditions less favorably. The two vulnerable cohorts who were less well adapted were late teen non-immigrant girls and early teen immigrant boys; the former reported experiencing higher daily stress and the latter reported having a weaker sense of belonging. For ongoing and future formation of policy, these findings suggest that prevention and intervention strategies need to be age and gender appropriate to assist adolescents’ well-being.

As the transition of health in immigrant acculturation process is the result of interactions between an individual process and a supra-individual process, [38] and consequential health outcomes are the result of interactions between individual compositional factors and societal contextual factors, [1] it is critical to characterize the immigrants’ experiences in their acculturation setting within a national society [62]. Canada is a country with one of the strongest multiculturalism policies. As specified in The Canadian Constitution Act 1982 and Charter of Rights and Freedoms, the multicultural heritage and ethnic diversity of Canadians as well as minority rights are officially recognized and guaranteed. Such official policies and societal environments might promote immigrants’ positive perception of own well-being with feeling welcomed and accepted as newcomers to Canada.

Considering that most immigrants to Canada are from collectivistic cultures, the ethnocultural background of immigrant adolescents can impact substantially on interactions with different sets of sociocultural rules which apply less restrictive boundaries for individuals [29,30]. As their length of residence prolongs, they will undergo gradual changes in acceptance of new social rules and in self-perceptions of own well-being by utilizing different ‘reference groups’ to shift from those of the country of original to those of the new settlement society. Identification of such reference groups for specific contexts was not possible with the data source for this study; information about the country of origin and exact length of residence were also not available. The measurement of length of residence by only two categories did not adequately detect developmental changes taking place among immigrant adolescents.

It can be argued that the immigrant adolescents examined in this study displayed positive outcomes due to the recency effect following their migration. After all, their length of residence was still relatively short. Moreover, their acculturation process had been supported by school systems and peer relationships in the learning environments. Consequently, they may have felt protected and optimistic about the social surroundings around them. That is, as the result of both institutional supports and personal social networks provided, they had opportunities to build psychological stability and confidence which is the cumulative consequence over time [33,31]. More studies are needed to examine the extent to which immigrant adolescents’ support network in their immediate social surroundings, such as friendship and mentor relations, benefit and facilitate their health and adaptation. In future research, these kinds of multiple-level effects can be further analyzed by structural equation modelling to specify mediating and moderating factors for adolescent well-being.

Some discussions may be also warranted to explain the differences found between the two immigrant populations, adult population samples in Canada [5] and the adolescent population in this study, since immigrant adults are not as well-adapted even though both groups are better in their health conditions than their respective counterparts of non-immigrants. In explaining the difference between the two cohorts, it would be appropriate to address to what extent social capitals and resources are available and accessible to them for their adaptation processes.

According to resource-based theoretical approaches, adolescent immigrants were supported by structural resources through institutionalized schooling which allow them to have access towards the new society while maintaining personal cultural resources through family and home contexts. In turn, their growing cultural knowledge and norms of the new society enable them to build efficient functional social networks without losing support from their own culture. As the adaptation for immigrants is to possess adequate abilities for the present surroundings as well as for the future gains seeking personal satisfaction and pursuing goals to achieve mobility in the various contexts of the new settlement, [32,31] immigrant adolescents in the process of cross-cultural acculturation seem to be better equipped than adult immigrants who are unfamiliar or less confident with such resources.

Further, the adaptation of adult immigrants depends on their own ability to regain lost resources by migration and to build new ones which would benefit them in the new country [31]. In their attempts to build or join new social networks, adult immigrants are bounded by the social boundaries formed by ethnicity and culture of origin, both of which demand solidarity and group-membership [63]. Their primary source of resources comes from their own ethnocultural communities already established in the settlement society because they provide a sense of embeddedness [63] and ethnic consciousness for them [32]. However, their own ethnocultural communities can also be a source of conflicts for individual immigrants who need to incorporate with and penetrate the new society by constraining their individual freedom [63] or by increasing social marginalization [32].

With respect to the limitations of this study, the present findings in support of the healthy immigrant effect do not exclude difficulties immigrants face in their settlement process. The current results do not predict the trajectory of immigrant health, [1] or the effect of national origin, [18] or the effect of the migrant community size, [62] or the influence by the specific immigrant status [57]. Attention also needs to be paid to the bases of the immigrant adolescent self-reports on their own health. Immigrant adolescents could have been influenced by their different expectations compared to their non-immigrant counterparts [64]. Their reports may vary across ethnocultural groups in assessment of health conditions and health behaviors, [65] and by willingness to report psychological and mental illness in particular [2,66,67]. Regarding the stressors related to daily stress level and somatization, it is conceivable that immigrant and non-immigrant adolescents have differential problems in their familial contexts [19,30,61]; so, the stressful impacts and possible coping strategies available to adolescents would also have been different.

In conclusion, the present study does provide evidence for healthy and well-adapted immigrant adolescent population samples in Canada. According to their self-report, immigrant adolescents to Canada were in better physical and psychological health, and were experiencing good adaptation equal to their native-born peers despite coming from a less affluent family background. This set of outcomes calls for further research which would promote and sustain the long-term good health of the immigrant population, in addition to the study of the social determinants on health and adaptation, in order to provide information with respect to the effectiveness of services available to various age-groups of immigrants, the prevention strategies for sustainability of physical and mental health, as well as the development of adequate immigration policies.
